# Hypochlorhydria‐induced calcium malabsorption does not affect fracture healing but increases post‐traumatic bone loss in the intact skeleton

**DOI:** 10.1002/jor.23221

**Published:** 2016-03-14

**Authors:** Melanie Haffner‐Luntzer, Aline Heilmann, Verena Heidler, Astrid Liedert, Thorsten Schinke, Michael Amling, Timur Alexander Yorgan, Annika vom Scheidt, Anita Ignatius

**Affiliations:** ^1^ Institute of Orthopedic Research and BiomechanicsUniversity Medical Center UlmHelmholtzstraße 14Ulm89081Germany; ^2^ Department of Osteology and BiomechanicsUniversity Medical Center Hamburg‐EppendorfMartinistraße 52Hamburg20246Germany

**Keywords:** fracture healing, hypochlorhydria, calcium malabsorption, calcium supplementation, post‐traumatic bone loss

## Abstract

Efficient calcium absorption is essential for skeletal health. Patients with impaired gastric acidification display low bone mass and increased fracture risk because calcium absorption is dependent on gastric pH. We investigated fracture healing and post‐traumatic bone turnover in mice deficient in *Cckbr*, encoding a gastrin receptor that affects acid secretion by parietal cells. *Cckbr*−/− mice display hypochlorhydria, calcium malabsorption, and osteopenia. *Cckbr*−/− and wildtype (WT) mice received a femur osteotomy and were fed either a standard or calcium‐enriched diet. Healed and intact bones were assessed by biomechanical testing, histomorphometry, micro‐computed tomography, and quantitative backscattering. Parathyroid hormone (PTH) serum levels were determined by enzyme‐linked immunosorbent assay. Fracture healing was unaffected in *Cckbr*−/− mice. However, *Cckbr*−/− mice displayed increased calcium mobilization from the intact skeleton during bone healing, confirmed by significantly elevated PTH levels and osteoclast numbers compared to WT mice. Calcium supplementation significantly reduced secondary hyperparathyroidism and bone resorption in the intact skeleton in both genotypes, but more efficiently in WT mice. Furthermore, calcium administration improved bone healing in WT mice, indicated by significantly increased mechanical properties and bone mineral density of the fracture callus, whereas it had no significant effect in *Cckbr*−/− mice. Therefore, under conditions of hypochlorhydria‐induced calcium malabsorption, calcium, which is essential for callus mineralization, appears to be increasingly mobilized from the intact skeleton in favor of fracture healing. Calcium supplementation during fracture healing prevented systemic calcium mobilization, thereby maintaining bone mass and improving fracture healing in healthy individuals whereas the effect was limited by gastric hypochlorhydria. © 2016 Orthopaedic Research Society. Published by Wiley Periodicals, Inc. J Orthop Res 34:1914–1921, 2016.

Because gastric calcium solubility is pH‐dependent,^1^, ^2^ impaired gastric acidification induces calcium malabsorption, which negatively affects bone properties. Therefore, patients with hypochlorhydria, which is defined by reduced stomach acid production due, for example, to the long‐term use of proton‐pump inhibitors (PPI), display a higher prevalence for osteoporosis and increased fracture risk.^3^, ^4^ Additionally, gastrectomized patients with achlorhydria display increased bone turnover and mineralization defects.^5^, ^6^, ^7^ This was confirmed experimentally in *Cckbr*−/− mice mimicking gastric hypochlorhydria.^8^ These mice are deficient in the cholecystokinin B‐gastrin receptor (Cckbr) that stimulates gastric acid secretion by parietal cells by binding the endogenous peptide hormone gastrin.^9^, ^10^ *Cckbr−/−* mice display gastric hypochlorhydria and calcium malabsorption. The resultant calcium deficiency leads to secondary hyperparathyroidism and excessive bone resorption, causing an osteoporotic bone phenotype.^8^ These results are confirmed by the osteoporotic bone phenotype in *Atp4b^−/−^* mice that lack the H^+^–K^+^‐ATPase on parietal cells required for gastric acid production.^7^, ^11^ Calcium supplements with solubility at neutral pH, such as calcium gluconate, are able to abolish increased bone resorption in both hypochlorhydria mouse models and in gastrectomized individuals.^7^, ^8^ Taken together, these findings demonstrate that functional gastric integrity that ensures intestinal calcium absorption plays a critical role in maintaining skeletal health.

An adequate calcium supply is also required for fracture healing, being essential for callus mineralization and bony bridging of the fracture gap.^12^, ^13^, ^14^ However, to our knowledge, the effect of gastric hypochlorhydria on fracture healing has not been investigated. This is striking, because >20% of osteoporotic fracture patients are treated with PPIs to reduce the risk for gastrointestinal side effects of bisphosphonates, which are commonly prescribed to prevent secondary osteoporotic fractures.^15^

Therefore, the aim of this study was to investigate the effect of calcium malabsorption and supplementation on fracture healing and post‐traumatic bone turnover in *Cckbr*−/− mice, which mimic gastric hypochlorhydria.

## MATERIALS AND METHODS

### Mice

All experiments were performed according to international regulations for the care and use of laboratory animals and were approved by the responsible Ethical Committee (No. 1026, Regierungspräsidium Tübingen, Germany). Female wildtype (WT) mice (129S6/SvEvTac) aged 26 weeks were obtained from Taconic Farms, Inc. (Taconic Farms Inc, NY). Female *Cckbr*−/− mice of the same age were provided by the University Medical Center Hamburg‐Eppendorf.^10^ All mice were housed in cages in maximal groups of four animals and kept on a 14‐h light and 10‐h dark rhythm, with water and food ad libitum. Until the day of surgery, all mice received the same standard diet containing 1% calcium from mixed conjugates (R/M‐H, V1535‐300, Ssniff Spezialitäten GmbH, Soest, Germany). After surgery, the mice were randomized in two groups, either receiving the standard diet (S) or a custom‐made diet (C1032, modified, Altromin Spezialfutter GmbH & Co. KG, Lage, Germany) enriched with an extra amount of 0.8% calcium gluconate (C). We chose calcium gluconate supplementation because of a greater solubility at neutral pH than other calcium conjugates.

### Surgical Procedure

All procedures were performed in mice anesthetized using 2% isoflurane (Forene, Abbott, Wiesbaden, Germany). The mice received a standardized osteotomy at the mid‐shaft of the right femur as described previously.^16^, ^17^, ^18^ Briefly, an osteotomy gap was created using a wire saw (diameter 0.4 mm, RISystem, Davos, Switzerland) and stabilized using an external fixator (axial stiffness 3.0 N/mm, RISystem). One day preoperatively until 3 days postoperatively, 25 mg/L tramalhydrochloride (Tramal, Gruenenthal GmbH, Aachen, Germany) was administered in drinking water as analgesic. One subcutaneous injection of clindamycin‐2‐dihydrogenphosphate (45 mg/kg, Clindamycin, Ratiopharm, Ulm, Germany) before surgery was used for antibiosis. Mice were sacrificed at 10, 24, or 32 days using cervical dislocation under general anesthesia.

### Serum Analysis

Before surgery and on the day of sacrifice, blood samples were obtained from the vena facialis. Serum PTH concentrations were determined using an enzyme‐linked immunosorbent assay (ELISA) kit according to the manufacturer's instructions (Mouse PTH 1‐84 ELISA Kit 60‐2305, Immutopics Inc., San Clemente, CA). Optical density of the samples was determined against standards with defined PTH concentrations using a microplate reader (Infinite M200 NanoQuant, Tecan Trading AG, Männedorf, Switzerland; Software Magellan Version 6).

### Biomechanical Testing

For biomechanical analysis of the intact and osteotomized femora, both femora were explanted at day 32 and tested by a nondestructive, three‐point‐bending test as described previously.^16^, ^17^, ^18^ Briefly, after removing the fixator, the proximal end of the femur was fixed using a two‐component adhesive (i‐Cem Self‐Adhesive, Heraeus Kulzer, Hanau, Germany) in an aluminum cylinder. The cylinder was fixed in a materials testing machine (Z10, Zwick Roell, Ulm, Germany). The femoral condyles rested unfixed on the distal bending support. Bending load was applied on top of the callus tissue with a maximum load of 4 N. Flexural rigidity was calculated from the linear elastic part of the load‐displacement curve.

### Micro‐Computed Tomography (µCT) Analysis

After biomechanical testing, femora were imaged using a µCT device (Skyscan 1172, Kontich, Belgium) at a resolution of 8 µm and a voltage of 50 kV and 200 µA. To determine the bone mineral density (BMD), two phantoms with a defined density of hydroxylapatite (250 mg/cm^3^ und 750 mg/cm^3^) were used for calibration. The threshold 642 mg/cm^3^ according to Morgan et al.^19^ was used to distinguish between mineralized and nonmineralized tissue. Using μCT analysis software (CTAnalyser, Skyscan), the region of the former osteotomy gap including periosteal and endosteal callus was defined as the volume of interest (VOI) for fracture healing analysis at day 32, because we were interested in the final outcome of bone healing. According to the standard clinical assessment of X‐rays, the number of bridged cortices per callus was evaluated in two perpendicular planes from μCT analysis. A “healed fracture” was considered to have ≥3 bridged cortices per callus. In the lumbar vertebrae L3‐4, a VOI of 8 mm diameter was used to examine the intact trabecular bone at day 24, because this time point was assumed to be the peak of post‐traumatic bone turnover in the intact skeleton.

### Histology

Fractured femora were processed for decalcified histology 10 days after surgery. Bones were fixed in 4% formalin for a minimum of 48 h and decalcified using 20% ethylenediaminetetraacetic acid (pH 7.2–7.4) for 8–14 days. After dehydration, femora were embedded in paraffin and 7‐µm longitudinal slices cut and stained using Safranin‐O for tissue quantification. The relative amounts of total osseous tissue, cartilage, and fibrous tissue were determined in the whole callus between the inner pin holes of the fixator, excluding cortical bone. For evaluation of the intact trabecular bone after 24 days, lumbar vertebrae L3–4 were used for undecalcified histology. They were fixed in 4% formalin for at least 48 h and dehydrated using increasing ethanol concentrations. After embedding in methyl methacrylate, 7‐µm slices were cut and stained with Giemsa to count the number of osteoclasts and osteoblasts under light microscopy (Leica DMI6000, Leica, Heerbrugg, Switzerland; Software MMAF Version 1.4.0 MetaMorph, Leica, Switzerland) at 50‐fold magnification. Criteria for osteoblast count were location at the bone surface, appropriate morphology and staining. Criteria for osteoclast count were location at the bone surface, more than three nuclei and appropriate morphology. One slice taken from the center of the callus was analyzed per mouse for each outcome parameter.

### Quantitative Backscattering

Quantitative backscattered electron imaging (qBEI) was used to determine the degree of mineralization of the fracture callus according to others.^20^, ^21^, ^22^ Briefly, methyl‐methacrylate‐embedded 4‐μm longitudinal sections from the fracture calli at day 24 were polished and carbon coated to determine the BMD distribution. The scanning electron microscope (LEO 435 VP, LEO Electron Microscopy Ltd., Cambridge, England) was operated at 15 kV and 665 pA at a constant working distance (BSE Detector, Type 202, K.E. Developments Ltd., Cambridge, England), with a 3‐μm pixel size. Synthetic hydroxyapatite samples (DOT Medical Solutions, Rostock, Germany) were used to create a calibration curve and contained different Ca/P ratios, which were determined by energy dispersive X‐ray analysis (DX‐4, EDAX, Mahwah, NJ) and qBEI. The generated gray values represent the mean calcium content (Ca mean wt%), peak calcium content (Ca peak wt%) and the heterogeneity of mineralization (width Ca width wt%) of the cancellous bone in the fracture callus.

### Statistical Analysis

All graphical values are presented as box plots with median, minimum, maximum, and interquartile ranges. Outliers (value is less than the first quartile minus 1.5 times the interquartile range or greater than the third quartile plus 1.5 times the interquartile range) were marked as circles. Statistics software IBM SPSS Statistics 21 (SPSS Inc., Chicago, IL) was used. Data were analyzed using Kruskal–Wallis test with Bonferroni correction, if more than two groups were compared to each other. Mann–Whitney *U*‐tests were used to determine the statistical significance, if only two groups were compared to each other. The level indicating significance was *p* ≤ 0.05. *n* = 5–12. Sample size was calculated based on a previous study for the main outcome parameter flexural rigidity of fractured femora (power 80%, alpha 0.05).^18^ The sample size for each experiment is indicated in the figures and tables.

## RESULTS

### Fracture Healing Was Unaffected in *Cckbr*−/− Mice

**Figure 1:**
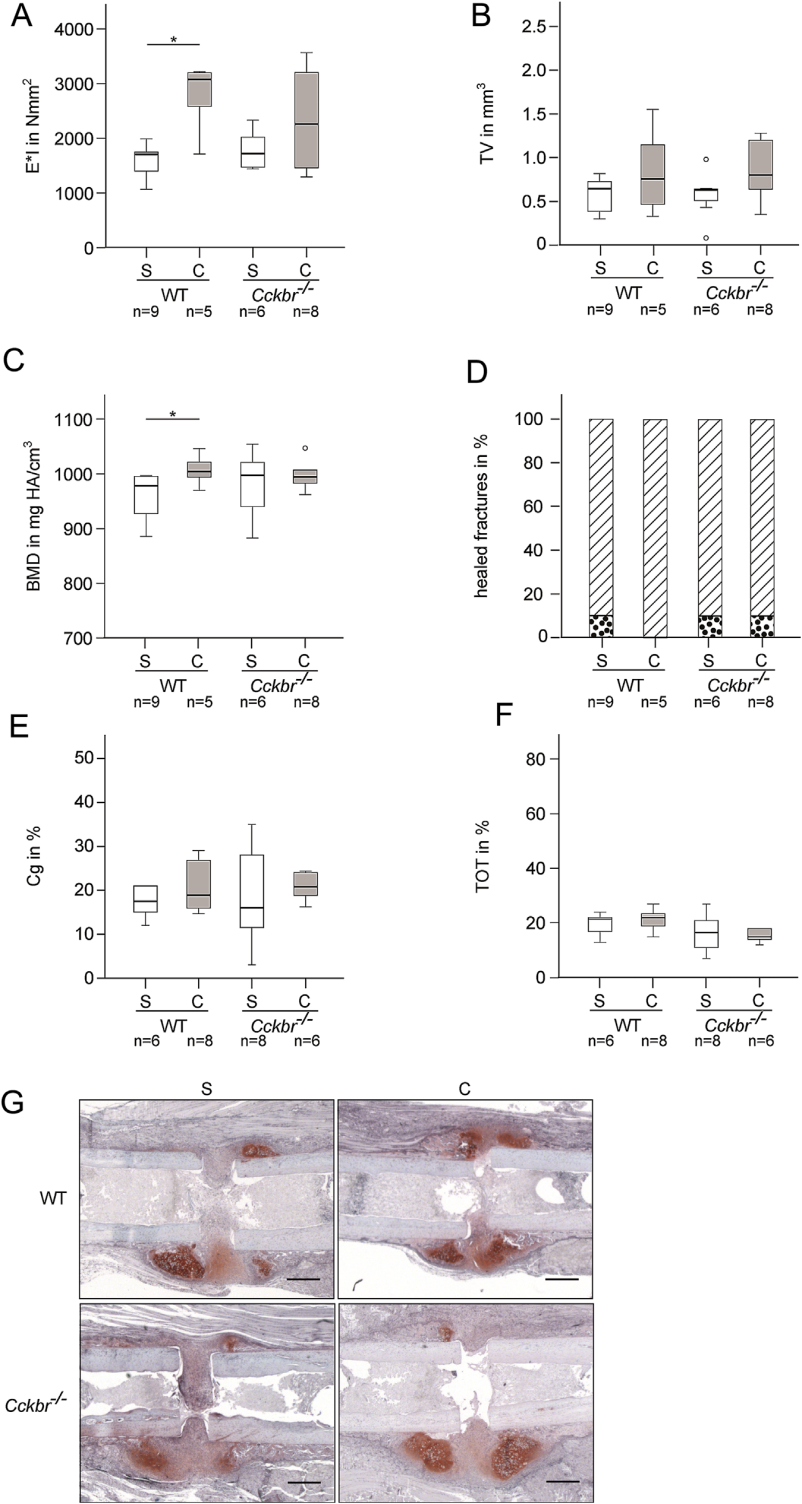
(A) Flexural rigidity of the fractured femora at day 32 of wildtype (WT) and *Cckbr*−/− mice fed with standard died (S) or supplemented with calcium (C). (B) Total callus volume (TV) and (C) bone mineral density (BMD) of the fracture callus at day 32. (D) Percentage of nonhealed (dotted bar) and healed fractures (hatched bar) per group. Healed fractures were defined as ≥3 bridged cortices. (E) Percentage of osseous tissue (TOT) and (F) cartilaginous tissue (Cg) in the fracture callus after 10 days. *vs. S, *p* ≤ 0.05. The sample size for each experiment is indicated below each group. (G) Representative images of the fracture callus at day 10 of WT and *Cckbr*−/− mice fed with standard died (S) or supplemented with calcium (C). Slices were stained with Safranin O to identify cartilage (red), bone (light blue), and fibrous tissue (purple). Scale bar: 500 μm.

Hypochlorhydria in *Cckbr*−/− mice did not cause impaired bone healing. Flexural rigidity, total volume, BMD, and bony bridging of the fracture callus were not significantly altered compared to WT mice (Fig. 1A–D). Moreover, qBEI analysis revealed that the amount and distribution of calcium in the newly formed bone were unaffected by calcium malabsorption (Table 1). We also analyzed the fracture calli histologically at an earlier time point (day 10) to assess endochondral bone formation. *Cckbr*−/− mice did not display differences in the relative amounts of bone and cartilage, indicating undisturbed cartilage formation and cartilage‐to‐bone transformation (Fig. 1E–G).

**Table 1:** Quantitative Backscattering Analyses of the Newly Formed Bone in the Fracture Callus of Wildtype (WT) and *Cckbr*−/− Mice at 24 Days Post Fracture

Parameters		WT S *n* = 6	WT C *n* = 5	*Cckbr*−/− S *n* = 5	*Cckbr*−/−C *n* = 6
Mean calcium	Ca mean in wt%	23.53 ± 0.58	25.28 ± 0.79^a^	23.54 ± 0.76	23.74 ± 0.77
Calcium peak	Ca peak in wt%	25.04 ± 0.79	28.38 ± 0.80^a^	25.63 ± 1.38	25.92 ± 0.34
Calcium width	Ca width in wt%	4.30 ± 0.35	4.90 ± 0.47^a^	4.54 ± 1.10	4.55 ± 0.55

S, standard diet; C, calcium‐supplemented diet.

Data are presented as the mean ± standard deviation. Level of significance *p* ≤ 0.05. The sample size for each experiment is indicated below each group.

#vs *Cckbr*−/− S, Level of significance *p* ≤ 0.05.

^a^ vs WT S, *p* ≤ 0.05.

### *Cckbr*−/− Mice Displayed Increased Calcium Mobilization From the Intact Skeleton After Fracture

**Figure 2:**
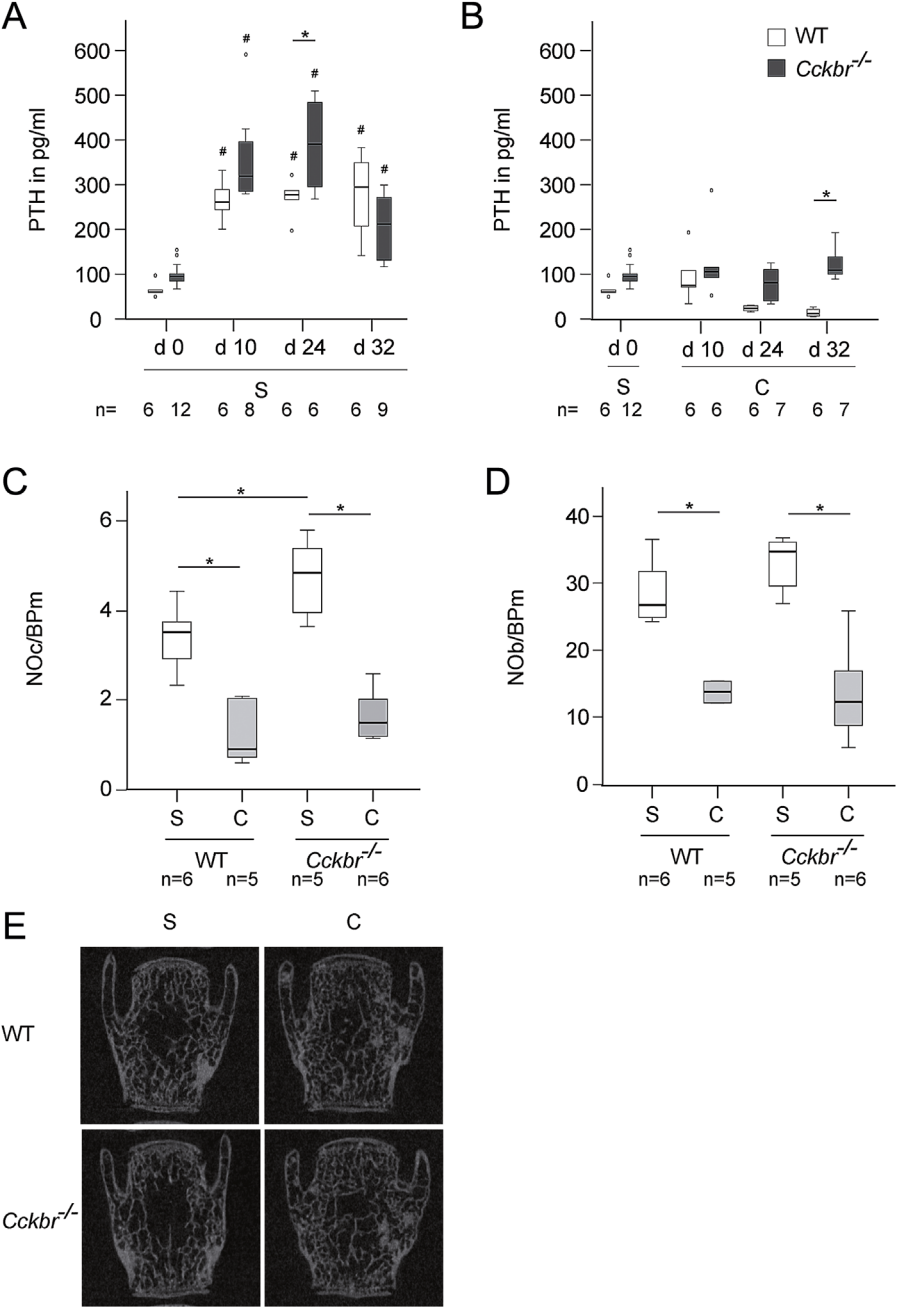
(A) Parathyroid hormone (PTH) levels in the serum of wildtype (WT) (white bars) and *Cckbr*−/− (dark gray bars) mice pre‐osteotomy (d0) and at days 10, 24, and 32 after osteotomy fed with standard diet (S). ^#^vs. d0 of the same genotype, *p* ≤ 0.05. (B) Serum PTH levels of WT (white bars) and *Cckbr*−/− mice (dark gray bars) pre‐osteotomy (d0) and at days 10, 24, and 32 after osteotomy fed with S until day 0 and with calcium supplementation (C) after osteotomy. ^#^vs. d0 of the same genotype, *p* ≤ 0.05, *vs. WT, *p* ≤ 0.05. (C) Number of osteoclasts per bone perimeter (NOc/BPm) and D) number of osteoblasts per bone perimeter (NOb/BPm) in the vertebral bodies at day 24 post fracture. *vs. WT or *Cckbr*−/− S, *p* ≤ 0.05. The sample size for each experiment is indicated below each group. (E) Representative micro‐computed tomography images from the vertebral body L4 at day 24 post fracture.

To assess calcium mobilization from the intact skeleton, we first analyzed PTH serum levels 10, 24, and 32 days after fracture. PTH levels after osteotomy significantly increased in both WT and *Cckbr*−/− mice compared to the pre‐osteotomy values (Fig. 2A). At day 24, *Cckbr*−/− mice displayed significantly higher PTH levels compared to WT mice. Because PTH increases osteoclast activity, we analyzed cell numbers and structural parameters of the nonfractured bone by histomorphometry and μCT. Indeed, the number of osteoclasts was significantly increased in the vertebral bodies of *Cckbr*−/− mice, whereas the number of osteoblasts did not show significant difference compared to WT mice (Fig. 2C and D). μCT analysis demonstrated no significant differences in BMD and structural parameters in the vertebral bodies (Table 2). However, biomechanical testing of the intact femur revealed a slightly decreased bending stiffness in the *Cckbr*−/− mice at day 32 (Table 2).

**Table 2:** Biomechanical and Micro‐Computed Tomography Analyses of the Intact Femora and the Trabecular Bone of L3–L4 at Day 24 Post Fracture of Wildtype (WT) and *Cckbr*−/− Mice Fed With Standard Diet (S) or Supplemented With Calcium (C)

Parameters		WT S *n* = 6	WT C *n* = 5	*Cckbr*−/− S *n* = 5	*Cckbr*−/− C *n* = 6
Femur					
Flexural rigidity	E*I in Nmm^2^	3197 ± 295	3293 ± 515	2770 ± 358^a^	3440 ± 474^b^
Cortical thickness	Ct.Th in mm	0.168 ± 0.006	0.166 ± 0.001	0.164 ± 0.002	0.166 ± 0.001^b^
Vertebral bodies					
Bone mineral density	BMD in mg/cm^3^	940 ± 9	1050 ± 25^a^	909 ± 30	987 ± 8^b^
Bone volume	BV/TV in %	20 ± 3	32 ± 3^a^	19 ± 4	29 ± 2^b^
Trabecular thickness	Tb.Th in mm	0.050 ± 0.002	0.063 ± 0.004^a^	0.047 ± 0.003^a^	0.057 ± 0.002^b^
Trabecular number	Tb.N in 1/mm	4.0 ± 0.4	5.0 ± 0.4^a^	4.0 ± 0.6	5.0 ± 0.5^b^
Trabecular separation	Tb.Sp in mm	0.229 ± 0.171	0.197 ± 0.040	0.221 ± 0.028	0.192 ± 0.025

Data are presented as the mean ± standard deviation. Level of significance *p* ≤ 0.05. The sample size for each experiment is indicated below each group.

^a^ vs WT S, *p* ≤ 0.05.

^b^ vs *Cckbr*−/− S, *p* ≤ 0.05.

### Calcium Supplementation Improved Bone Healing in WT but Not in *Cckbr−/−* Mice

We next addressed the question whether calcium supplementation improves fracture healing. Calcium supplementation had a positive effect on bone healing in WT mice, whereas fracture healing was not significantly altered in *Cckbr*−/− mice. Fractured femora of WT mice displayed a significantly increased flexural rigidity and BMD after calcium supplementation at day 32 (Fig. 1A and C). Total callus volume (Fig. 1B) and the amount of cartilaginous and bony tissue (Fig. 1E and F) in the fracture callus did not show significant differences at day 10. However, quantitative backscattering analysis indicated a significantly increased amount of calcium in the newly formed bone in supplemented WT mice, but not in supplemented *Cckbr*−/− mice (Table 1).

### Post‐Traumatic Calcium Mobilization in *Cckbr−/−* Mice Was Ameliorated by Calcium Supplementation

Because *Cckbr*−/− mice fed with standard diet displayed increased PTH levels during fracture healing in comparison to WT mice, we also analyzed PTH serum levels after calcium supplementation. In both WT and *Cckbr*−/− mice, the strong post‐traumatic PTH increase was abolished by calcium supplementation (Fig. 2B); being similar to the pre‐osteotomy values at all time points (days 10, 24, and 32). The PTH decrease was more efficient in WT mice. The number of osteoclasts and osteoblasts were significantly reduced in the trabecular bone of the vertebral bodies of both WT and *Cckbr*−/− mice fed with calcium‐enriched diet (Fig. 2C and D). Furthermore, in both WT and *Cckbr*−/− mice, BMD, bone volume to tissue volume, trabecular thickness, and trabecular number were significantly increased after calcium supplementation (Table 2, Fig. 2E). Biomechanical testing and μCT analysis of the intact femora demonstrated that the reduced bending stiffness of the intact femora in *Cckbr*−/− mice 32 days after osteotomy was abolished by calcium supplementation. Cortical thickness was significantly increased in *Cckbr*−/− mice after supplementation (Table 2).

## DISCUSSION

Due to the increasing prevalence of patients with hypochlorhydria^23^ and the high rate of osteoporotic fracture in patients treated with PPIs,^15^ it is of considerable clinical relevance to elucidate the effect of calcium malabsorption on fracture healing. Notably, to date this has not been investigated, although it is generally accepted that calcium is essential for callus mineralization.^12^, ^13^, ^14^ There are only a few studies showing a moderate effect of dietary calcium‐deficiency on bony callus development.^24^, ^25^ Therefore, the aim of this study was to investigate fracture healing in *Cckbr*−/− mice, which display lower levels of gastric acid, impaired intestinal calcium absorption, and an osteoporotic bone phenotype.^8^ Notably, we found that calcium malabsorption did not significantly affect fracture healing in *Cckbr*−/− mice. However, these mice displayed increased PTH serum levels after fracture, which is an indicator for calcium deficiency and leads to increased bone resorption to restore physiological calcium concentrations.^26^, ^27^, ^28^, ^29^ Therefore, we suggested that *Cckbr*−/− mice displayed increased calcium mobilization in the intact skeleton after fracture compared to WT mice because of their impaired gastric calcium solubility. The significantly increased number of osteoclasts in the intact skeleton and the decreased flexural rigidity of the intact femur confirm this assumption. It has been shown previously in both experimental and clinical studies that fracture events may result in increased post‐traumatic calcium mobilization and bone loss in the intact skeleton, particularly in osteoporotic or vitamin D‐deficient patients.^14^, ^30^, ^31^, ^32^, ^33^, ^34^, ^35^ PTH serum levels of elderly fracture patients remained elevated 1 year after fracture, indicating increased calcium mobilization from the intact skeleton during a long period after the initial injury.^35^ The post‐traumatic bone loss after initial fracture may cause the threefold higher risk for further fractures reported in clinical studies.^36^, ^37^

In the present study, we also found significantly elevated serum PTH levels in WT mice, indicating that the normal standard diet may contain insufficient amounts of available calcium for fracture healing. Therefore, calcium mobilization from the skeleton was also increased in WT mice after fracture, but due to their normal gastric calcium solubility and absorption, the effects were minor compared to *Cckbr*−/− mice.

One strategy against post‐traumatic bone loss may be calcium supplementation. Therefore, we investigated whether calcium supplementation using calcium gluconate, which has a greater solubility at neutral pH than other calcium conjugates, influences fracture healing in both WT and *Cckbr*−/− mice. Long‐term supplementation with calcium gluconate has been shown to reverse the osteoporotic phenotype of *Cckbr*−/− mice.^8^ Additionally, Atp4d‐deficient mice fed with calcium gluconate for 4 weeks displayed decreased PTH levels and osteoclast numbers compared to mice fed with standard diet.^7^ In the present study, calcium supplementation improved fracture healing in WT, but not in *Cckbr*−/− mice. The BMD and calcium content in the fracture callus was significantly increased in WT mice fed with calcium‐enriched diet, resulting in increased mechanical competence of the fractured bone. These results confirmed previous studies showing that dietary calcium supplementation increased bony bridging of the fracture gap in ovariectomized rats^38^ and that a single high‐dose injection of calcium increased the biomechanical properties of fractured tibias in non‐ovariectomized rats.^39^ Moreover, PTH serum levels did not increase after fracture in supplemented WT mice. These findings additionally underlined the hypothesis, that the standard diet might contain insufficient calcium for fracture healing. Supplemented mice did not need to mobilize calcium from the intact bones to allow normal fracture healing. Moreover, the number of osteoclasts in the intact skeleton was significantly decreased, resulting in increased BMD, trabecular thickness, and trabecular number in the vertebral bodies. Therefore, our study provides evidence that there may be positive effects of calcium uptake during fracture healing in patients with lower dietary calcium consumption not only on callus mineralization but also on BMD of the nonfractured bones.

Contrary to our expectations, the fracture healing process was not improved in *Cckbr*−/− mice by calcium gluconate supplementation. However, increased PTH levels after fracture were reduced. Therefore, calcium gluconate supplementation may have led to higher gastric calcium absorption in *Cckbr*−/− mice, resulting in ameliorated post‐traumatic calcium mobilization from the intact skeleton after fracture. The reduced osteoclast number in the intact trabecular bone in these mice underlined this conclusion. Moreover, supplemented *Cckbr*−/− mice displayed increased flexural rigidity and cortical thickness of the intact femur as well as increased BMD, trabecular thickness, and trabecular number in the vertebral bodies compared to *Cckbr*−/− mice fed with standard diet. However, supplemented *Cckbr*−/− mice still displayed significantly higher PTH levels than supplemented WT mice, which may explain why fracture healing was not improved in *Cckbr*−/− mice.

Clinical data showed that achlorhydria patients supplemented with calcium gluconate displayed increased BMD.^7^ However, there are no prospective studies where fractured achlorhydric or hypochlorhydric patients were treated with calcium gluconate supplementation during the healing period. An observational study showed an increased BMD in the intact tibia of a gastrectomized patient suffering from atraumatic forearm fractures and supplemented with calcium gluconate, indicating the strong clinical need for calcium gluconate supplementation in these patients after fracture to prevent post‐traumatic bone loss.^7^

In conclusion, the present study demonstrated that, under conditions of hypochlorhydria‐induced calcium malabsorption, calcium, which is essential for callus mineralization, is increasingly mobilized from the intact skeleton in favor of fracture healing. The post‐traumatic bone loss may be responsible for the increased risk for further fractures after an initial fracture event.^35^, ^37^ Calcium supplementation during fracture healing could prevent systemic calcium mobilization, thereby maintaining bone mass and even improve fracture healing in healthy individuals. In patients with gastric hypochlorhydria, it is important to apply a sufficient amount of calcium conjugates with high solubility at neutral pH, for example, calcium gluconate.

## AUTHORS’ CONTRIBUTIONS

Study design: AH, AI, TS, MA. Study conduct: MHL, AH. Data collection: MHL, AH, AvS. Data analysis: MHL, AH, AvS, TAY. Data interpretation: MHL, AH, VH, AI, TS, AvS. Drafting manuscript: MHL, VH, AI, AH. Revising manuscript content: MHL, AH, VH, AL, TS, MA, AvS, TAY, AI. Approving final version of manuscript: MHL, AH, VH, AL, TS, MA, TAY, AvS, AI.
